# High frequency jet ventilation through mask contributes to oxygen therapy among patients undergoing bronchoscopic intervention under deep sedation

**DOI:** 10.1186/s12871-021-01284-y

**Published:** 2021-03-02

**Authors:** Mingyuan Yang, Bin Wang, Qingwu Hou, Yunzhi Zhou, Na Li, Hongwu Wang, Lei Li, Qinghao Cheng

**Affiliations:** 1 Department of Anesthesiology, Emergency General Hospital, Beijing, 100028 China; 2 Department of Pulmonary and Critical Care Medicine, Emergency General Hospital, Beijing, China

**Keywords:** Bronchoscopic intervention, Conventional oxygen therapy, Normal frequency jet ventilation, High frequency jet ventilation

## Abstract

**Background:**

High frequency jet ventilation (HFJV) is an open ventilating technique to maintain ventilation for emergency or difficult airway. However, whether jet ventilation or conventional oxygen therapy (COT) is more effective and safe in maintaining adequate oxygenation, is unclear among patients with airway stenosis during bronchoscopic intervention (BI) under deep sedation.

**Methods:**

A prospective randomized cohort study was conducted to compare COT (high flow oxygen) with normal frequency jet ventilation (NFJV) and HFJV in oxygen supplementation during BI under deep sedation from March 2020 to August 2020. Patients receiving BI under deep sedation were randomly divided into 3 parallel groups of 50 patients each: the COT group (fractional inspired oxygen (FiO_2_) of 1.0, 12 L/min), the NFJV group (FiO_2_ of 1.0, driving pressure of 0.1 MPa, and respiratory rate (RR) 15 bpm) and the HFJV Group (FiO_2_ of 1.0, driving pressure of 0.1 MPa, and RR of 1200 bpm). Pulse oxygen saturation (SpO_2_), mean arterial blood pressure and heart rate were recorded during the whole procedure. Arterial blood gas was examined and recorded 15 min after the procedure was initiated. The procedure duration, dose of anesthetics, and adverse events during BI in the three groups were also recorded.

**Results:**

A total of 161 patients were enrolled, with 11 patients excluded. The clinical characteristics were similar among the three groups. PaO_2_ of the COT and NFJV groups was significantly lower than that of the HFJV group (*P* < 0.001). PaO_2_ was significantly correlated with ventilation mode (P < 0.001), body mass index (BMI) (*P* = 0.019) and procedure duration (*P* = 0.001). Multiple linear regression showed that only BMI and procedure duration were independent influencing factors of arterial blood gas PaO_2_ (*P* = 0.040 and *P* = 0.002, respectively). The location of airway lesions and the severity of airway stenosis were not statistically correlated with PaCO_2_ and PaO_2_.

**Conclusions:**

HFJV could effectively and safely improve intra-operative PaO_2_ among patients with airway stenosis during BI in deep sedation, and it did not increase the intra-operative PaCO_2_ and the risk of hypercapnia. PaO_2_ was correlated with ventilation mode, BMI and procedure duration. Only BMI and procedure duration were independent influencing factors of arterial blood gas PaO_2_. PaCO_2_ was not correlated with any preoperative factor.

**Trial registration:**

Chinese Clinical Trial Registry. Registration number, ChiCTR2000031110, registered on March 22, 2020.

## Background

Bronchoscopic intervention (BI) has become the preferred method for the diagnosis and treatment of airway lesions. It plays an important role in improving the patients’ quality of life, especially by relieving airway obstructions [1]. Different procedures require different methods of anesthesia and different airway management. Various oxygen delivery routes include nasal catheters, face masks, laryngeal mask airway (LMA) and supraglottic tubes, such as Wei nasal jet tube, endotracheal tube, and rigid bronchoscope [2, 3]. Endoscopic face mask is one of the most commonly-used oxygen-delivery devices for BI because of its high comfort and non-influence on bronchoscope insertion. Different ventilation modes, including conventional oxygen therapy (COT), intermittent ventilation, controlled mechanical ventilation, and jet ventilation could be utilized for conducting differentiated airway management, which have their own advantages and disadvantages [4–6].

Hypoxemia is a major complication and thus the main focus of anesthesia during deep sedation especially among patients undergoing BI [7, 8]. The main mechanisms of hypoxemia in BI are ventilation/blood flow imbalance and decreased ventilation in accordance with airway stenosis, especially after sedation. With the continuous development of modern medicine, the method for oxygen supply has changed, and the traditional oxygen therapy is no longer the classic and only method available. High-flow oxygen through nasal cannula or mask was used among patients infected with COVID-19 associated with severe hypoxemia, and it showed reductions in the need for therapeutic escalation and intubation among patients who received high-flow oxygen [9]. Supraglottic jet ventilation (SJV) is also superior to conventional means of oxygen delivery among patients with obesity who are under intravenous anesthesia [10].

High-frequency jet ventilation (HFJV) is believed to improve oxygenation of patients during BI, but whether jet ventilation is superior to COT and normal-frequency jet ventilation (NFJV) in maintaining intra-operative oxygenation is unclear among patients with airway stenosis during BI under deep sedation. Specially, we evaluated the hypothesis that HFJV could improve intra-operative PaO_2_ among patients with airway stenosis during BI in deep sedation.

## Methods

### Study design

This prospective randomized study aims to assess the efficacy and safety of oxygen supplementation via three different ventilation modes including COT, NFJV, and HFJV, during BI under deep sedation.

This study was approved by the Medical Research Ethics Committee of Emergency General Hospital in Beijing, China (K20–9). All the patients or their relatives signed informed consent prior to the commencement of the study program. This study was registered in Chinese Clinical Trial Registry on March 22, 2020 (Registration number: ChiCTR2000031110). It also followed the Consolidated Standards of Reporting Trials (CONSORT) guidelines.

### Study population

All patients were selected by the Center of BI, Emergency General Hospital, to undergo BI under deep sedation from March 2020 to August 2020. Inclusion criteria were as follows: (1) Scheduled for electric flexible bronchoscope; (2) Duration of operation, between 20 and 60 min; (3) Age, 18–80 years. Exclusion criteria were as follows: (1) Diagnosed with cardiac respiratory failure and coma; (2) T-tube, endotracheal intubation, and tracheotomy, or SpO_2_ < 90% in room air before the surgery; (3) History of mental and neurological disorders, sedative or hypnotic drugs and alcohol abuse; (4) Change of anesthesia method during the operation; (5) Intra-operative massive hemorrhage; (6) Patients transferred back to ICU with endotracheal intubation after operation.

In accordance with different ventilation modes, the eligible patients were randomized in a 1:1:1 ratio into three parallel groups by a physician unaware of the study. The three groups were the COT group, the NFJV group, and the HFJV group. To ensure blinding, the group allocation number was placed in an envelope, which the anesthesiologist opened preoperatively. A data investigator collected and recorded all perioperative data. The statistician and investigator were independent and blinded to the treatment.

BI procedures were advanced diagnostic and therapeutic procedures, which included laser, electrocautery, cryotherapy, balloon dilation, argon plasma coagulation and photodynamic therapy. All BI procedures were performed by experienced endoscopists using electric flexible bronchoscopy (Pentax, Japan).

### Anesthetic settings and maintenance

Upon the entrance to the operating room, patients underwent electrocardiogram (ECG), pulse oximetry (SpO_2_), and blood pressure monitoring. Lidocaine (1%, 10 ml) was administered via the spray-as-you-go technique before intervention. An endoscopic face mask was provided for oxygenation. The total bolus dose of remifentanil (40 μg·ml^− 1^) was 1 μg·kg^− 1^ and 1 ml was injected each time with an interval of 1 min. Then, propofol (1 mg·kg^− 1^) was injected 2 min after remifentanil was administered during the induction of anesthesia. Continuous injection of remifentanil (0.10 ~ 0.15 μg·kg^− 1^·min^− 1^) and propofol (30 ~ 50 μg·kg^− 1^·min^− 1^) by microinjection pumps was performed in accordance with the patient’s vital signs. The fluctuation of the patient’s mean arterial blood pressure (MAP) was controlled at 20% of the baseline. If the fluctuation of MAP was more than 20%, the depth of anesthesia was adjusted or vasopressor was given. The patients maintained a Ramsey sedation scale (RSS) score of 3 ~ 4. They breathed spontaneously during BI. When the SpO_2_ value was< 95%, the anesthesiologists increased the driving pressure by 0.05 MPa, and when the SpO_2_ value was< 90%, the anesthesiologists performed mask-bag ventilation.

The oxygen for COT was provided at fractional inspired oxygen (FiO_2_) of 1.0 with a flow rate of 12 L/min through a ventilator (Drager, Germany). The oxygen for NFJV was provided at FiO_2_ of 1.0 with a driving pressure (DP) of 0.1 MPa, respiratory rate (RR) of 15 bpm, and I/E ratio of 1:1.5 through jet ventilation (Twin Stream™, Austria). The oxygen for HFJV was provided at FiO_2_ of 1.0 with a DP of 0.1 MPa, RR of 1200 bpm and I/E ratio of 1:1.5 through jet ventilation (Twin Stream™, Austria).

### Data collection

The location and severity of airway stenosis were recorded by the data recorder before BI. The location of airway lesions was divided into four types: 1) upper and middle parts of the main airway, 2) middle and lower parts of the main airway, 3) left or right main bronchial, and 4) distal bronchial. The severity of airway stenosis was graded to three levels: 1 for 0% ~ 59%, 2 for 60% ~ 89%, and 3 for above 90%. The following parameters were continuously monitored during anesthesia: SpO_2_, MAP, heart rate (HR) and ECG. Meanwhile, the patients’ SpO_2_, MAP and HR were recorded at baseline (T_0_), the beginning of procedure (T_Beg_), 15 min after the initiation of procedure (T_15_), and the end of procedure (T_End_). Arterial blood gas (ABG) was examined and recorded 15 min after the initiation of procedure. The procedure duration and dose of anesthetics during BI in the three groups were also recorded. Adverse events including intra-operative hypoxemia (SpO_2_ < 90%), intra-operative hypercapnia (PCO_2_ ≥ 50 mmHg), intra-operative severe hypercapnia (PCO_2_ ≥ 100 mmHg), post-operative hypoxemia (SpO_2_ < 90%), post-operative hypercapnia (PCO_2_ ≥ 50 mmHg), post-operative severe hypercapnia (PCO_2_ ≥ 100 mmHg), and post-operative agitation, were recorded by data investigator. The primary outcomes were PaO_2_ among three groups and its influencing factors, and the secondary outcomes were PaCO_2_ among three groups and its influencing factors.

## Statistical analysis

PaO_2_ was seen as the primary outcome in this study. The mean ± standard deviation (SD) of PaO_2_ was 164.0 ± 73.4 mmHg, 220.2 ± 86.5 mmHg, and 210.2 ± 65.1 mmHg of the COT, NFJV and HFJV groups according to a pilot study of 5 patients, respectively. The sample size was estimated by the formula of n = (μ_α_ + μ_β_)^2^σ^2^/δ^2^with a standard deviation of 0.8, and bilaterally equal to 0.05, or even 0.2 (power = 0.8). We thus calculated to enroll 35 patients in each group.

SPSS 20.0 software was used for data collation and statistical analysis. The continuous data were expressed as mean ± SD, and the counting data were presented as the number and percentage. Chi-square test was used to compare the counting data of different groups (the *P* value was directly calculated using Fisher’s exact probability method if necessary). Univariate analysis of variance (ANOVA) was used for overall comparison among groups, and least significance difference (LSD) was used for multiple comparison between groups. Pearson analysis was used to investigate the correlation between blood gas indicators of PaO_2_ and PaCO_2_ and the clinical characteristics of patients. Multiple linear regression analysis was used to explore the independent influencing factors of PaO_2_ and PaCO_2_. *P* < 0.05 was considered statistically significant.

## Results

**Fig. 1:**
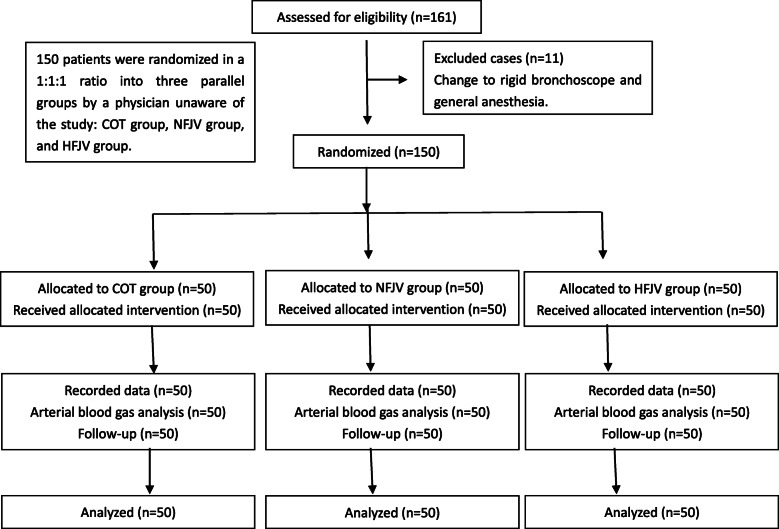
Diagram

A total of 161 patients were enrolled. Three patients in the COT group, four patients in the NFJV group and another four patients in the HFJV group were excluded because of change to rigid bronchoscope and general anesthesia during operation. All the remaining patients tolerated the BI well (Fig. 1). Two patients in the COT group (lowest SpO_2_ values were 79 and 85%, lasting for 2 min and 1 min), three patients in the NFJV group (lowest SpO_2_ values were 81, 83, and 87%, lasting for 1 min, 1 min, and 2 min, respectively) and two patients in the HFJV group (lowest SpO_2_ values were 87 and 88%, both lasting for 1 min) developed hypoxemia. No other adverse events such as severe hypercapnia and postoperative agitation occurred.

The clinical characteristics of patients are represented in Table 1. No significant differences were found among the three groups in terms of age, height, weight, body mass index (BMI), gender, pathological type, ASA classification, lesion site and stenosis degree. However, significant differences were observed among the three groups in terms of the following comorbidities: cerebrovascular disease, other tumors (such esophageal cancer and thyroid cancer), and chronic lung disease (including tuberculosis pneumonia, and COPD). The number of patients with cerebrovascular disease in the COT group was significantly higher than that in the NFJV and HFJV groups (*P* = 0.011). The number of patients with other tumors in the NFJV group was significantly higher than that in the HFJV group (*P* = 0.029). The number of patients with chronic lung disease in the COT group was significantly higher than that in the NFJV group (*P* = 0.017).

**Table 1:** Comparison of preoperative patient characteristics among three groups

Characteristic	Group COT(*n* = 50)	Group NFJV(n = 50)	Group HFJV(n = 50)	*P Value*
Age, mean ± SD, years	49.1 ± 14.4	54.2 ± 15.5	51.6 ± 14.4	0.227
Height,mean ± SD, (cm)	165.0 ± 7.7	167.2 ± 7.1	167.0 ± 8.7	0.316
Weight, mean ± SD, (Kg)	61.3 ± 8.3	63.5 ± 11.0	63.6 ± 10.3	0.423
Body Mass Index, mean ± SD(%)	22.6 ± 3.4	22.7 ± 3.4	22.7 ± 2.7	0.976
Male, n (%)	33 (66)	32 (64)	34 (68)	0.915
**ASA score**				0.157
ClassI, n (%)	0 (0)	0 (0)	2 (4)	
ClassII, n (%)	20 (40)	26 (52)	27 (54)	
Class III, n (%)	30 (60)	24 (48)	21 (42)	
**Comorbidities**				
Cardiovascular history, n (%)	9 (18)	12 (24)	11 (22)	0.757
Cerebrovascular disease, n (%)	5 (10)^bc^	0 (0)^a^	0 (0)^a^	**0.011**
Other tumors, n (%)	5 (10)	9 (18)^c^	1 (2)^b^	**0.029**
Diabetes mellitus, n (%)	3 (6)	6 (12)	10 (20)	0.108
Chronic pulmonary disease, n (%)	6 (12)^c^	0 (0)^a^	1 (2)	**0.017**
Tracheal esophageal fistula, n (%)	1 (2)	2 (4)	5 (10)	0.277
Lobectomy, n (%)	10 (20)	6 (12)	6 (12)	0.426
**Pathology**				
Non-tumor, n (%)	20 (40)	23 (46)	18 (36)	0.592
Tumor, n (%)	30 (60)	27 (54)	32 (64)	0.592
**lesion location**				0.074
Upper main airway, n (%)	17 (34)	14 (28)	10 (20)	
Lower main airway, n (%)	12 (24)	22 (44)	16 (32)	
Left and right bronchus, n (%)	8 (16)	10 (20)	9 (18)	
Others, n (%)	13 (26)	4 (8)	15 (30)	
**Degree of stenosis**				0.837
0–59%, n (%)	28 (56)	33 (66)	32 (64)	
60–89%, n (%)	12 (24)	10 (20)	9 (18)	
≥ 90%, n (%)	10 (20)	7 (14)	9 (18)	

Data were expressed as mean ± standard deviation or as numbers and percentages. ^a^ was statistically significant compared with COT group, ^b^ was statistically significant compared with HFJV group, ^c^ was statistically significant compared with NFJV group

The number of patients with cerebrovascular disease in the COT group was significantly higher than that in the NFJV and HFJV groups (*P* = 0.011). The number of patients with other tumors (including esophageal cancer, thyroid cancer, etc) in the NFJV group was significantly higher than that in the HFJV group (*P* = 0.029). The number of patients with chronic lung disease (including tuberculosis pneumonia, COPD, etc) in the COT group was significantly higher than that in the NFJV group (*P* = 0.017)

*COT* conventional oxygen therapy; *NFJV* normal frequency jet ventilation; *HFJV* high frequency jet ventilation

The blood gas values and procedure duration are shown in Table 2. PaO_2_, lactic acid and procedure duration significantly differed among the three groups (*P* < 0.001, *P* = 0.005, and *P* = 0.038, respectively). The PaO_2_ in the HFJV group was significantly higher than that in the COT and NFJV groups. Moreover, the lactic acid in the HFJV group was significantly higher and procedure duration was significantly longer. No significant differences in PaCO_2_, blood glucose and pondus hydrogenii (PH) were found among the three groups.

**Table 2:** Blood gas values and procedure duration among three groups

Patients values	Group COT(n = 50)	Group NFJV(*n* = 50)	Group HFJV(n = 50)	*P* Value
PaO_2_ (mmHg)	176.3 ± 73.4^b^	192.0 ± 88.4^b^	251.7 ± 86.6^ac^	**< 0.001**
PaCO_2_(mmHg)	59.2 ± 15.2	59.8 ± 14.1	59.6 ± 10.7	0.972
Pondus hydrogenii	7.3 ± 0.1	7.3 ± 0.1	7.3 ± 0.1	0.647
Glucose (mmol/l)	6.4 ± 1.8	6.9 ± 1.9	7.0 ± 2.1	0.203
Lactic acid (mmol/l)	1.1 ± 0.4^b^	1.4 ± 0.7	1.6 ± 1.1^a^	**0.005**
Procedure duration (min)	27.9 ± 8.8^b^	29.3 ± 8.5	33.0 ± 12.7^a^	**0.038**

*Data were presented as mean ± standard deviation (median, range). ^a^ was statistically significant compared with COT group, ^b^ was statistically significant compared with HFJV group, ^c^ was statistically significant compared with NFJV group

There were significant differences among the three groups in PaO_2_, lactic acid and procedure duration (*P* < 0.001, *P* = 0.005, *P* = 0.038). Moreover, PaO_2_ of HFJV group was significantly higher than that of COT and NFJV group. Lactic acid and procedure duration of HFJV group was significantly higher than that of COT group

*COT* conventional oxygen therapy; *NFJV* normal frequency jet ventilation; *HFJV* high frequency jet ventilation

**Fig. 2:**
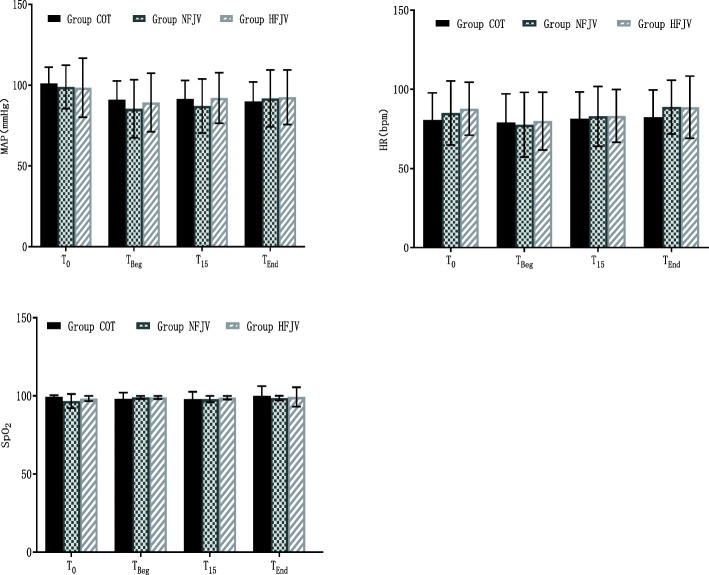
Hemodynamic indexes among three groups. No significant differences among three groups in MAP, HR and SpO_2_ at different time points. Patients’ SpO_2_, MBP and HR were recorded at baseline (T_0_), beginning of procedure (T_Beg_), 15 min after initiation of procedure (T_15_), and at the end of procedure (T_End_). COT: conventional oxygen therapy; NFJV: normal frequency jet ventilation; HFJV: high frequency jet ventilation

**Fig. 3:**
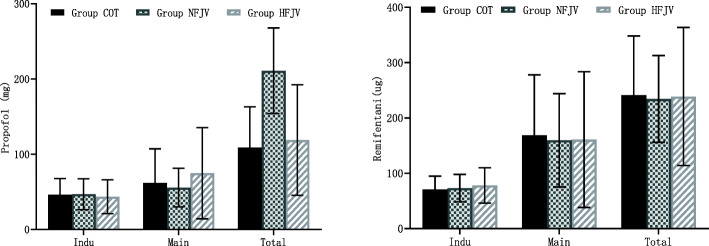
Anesthetic doses among the three groups. No significant differences among three groups in propofol and remifentanil at different periods. Induction (Indu), maintenance (Main) and total (Total) dose of anesthetics during bronchoscopic intervention in the three groups were recorded. COT: conventional oxygen therapy; NFJV: normal frequency jet ventilation; HFJV: high frequency jet ventilation

The MAP, HR, and SpO_2_ of each period time (Fig. 2) and anesthetic dose (Fig. 3) among the three groups did not significantly differ.

All peri-operative related factors associated with PaO_2_ were detected via Pearson analysis. The result showed that PaO_2_ was significantly correlated with ventilation mode (*P* < 0.001), BMI (*P* = 0.019) and procedure duration (*P* = 0.001). The ASA level, location of airway lesions, and severity of airway stenosis had no statistical correlation with PaO_2_. Multiple linear regression showed that BMI and procedure duration were independent influencing factors (*P* = 0.040, *P* = 0.002), while ventilation mode was not an independent factor. The details are shown in Tables 3 and 4.

**Table 3:** Pearson analysis of blood gas PaO_2_ related factors

Characteristic	F	*P*
Group COT/NFJV/HFJV	11.5	**< 0.001**
Body mass index	−0.2	**0.019**
Procedure duration	0.3	**0.001**
ASA score	2.0	0.143
Lesion location	2.2	0.092
Degree of stenosis	2.3	0.101

Pearson analysis showed that PaO_2_ was significantly correlated with ventilation mode, body mass index and procedure duration

*COT* conventional oxygen therapy; *NFJV* normal frequency jet ventilation; *HFJV* high frequency jet ventilation

Lesions location: 1) the upper and middle part of the main airway, 2) the middle and lower part of the main airway, 3) the left or right main bronchial and 4) in the distal bronchial. Degree of stenosis: 1 for 0–59%, 2 for 60–89%, and 3 for above 90%

**Table 4:** Multiple linear regression analysis of blood gas PaO_2_ related factors

Variables	Regression coefficient	95% Confidence interval	*P* Value
Body mass index	−4.6	(−9.0, −0.2)	**0.040**
Procedure duration	2.1	(0.8, 3.5)	**0.002**
Group COT/NFJV/HFJV	6.5	(−10.3, 23.3)	0.447

Multiple linear regression results showed that body mass index and procedure duration were independent influencing factors of blood gas PaO_2_ respectively

*COT* conventional oxygen therapy; *NFJV* normal frequency jet ventilation; *HFJV* high frequency jet ventilation

All the factors associated with PaCO_2_ were also detected via Pearson analysis, as shown in Table 5. No statistical correlation was found between blood gas PaCO_2_ and ventilation mode, lesion location, stenosis degree, and procedure duration. Multiple linear regression demonstrated that the above indices were not independent influencing factors of blood gas PaCO_2_, as shown in Table 6.

**Table 5:** Pearson analysis of blood gas PaCO_2_ related factors

Characteristic	F	P
Group COT/NFJV/HFJV	< 0.1	0.972
Body mass index	0.1	0.351
Procedure duration	< 0.1	0.601
ASA score	0.5	0.616
Lesion location	0.2	0.869
Degree of stenosis	0.7	0.491

Pearson analysis showed that there was no statistical correlation between blood gas PaCO_2_ and ventilation mode, ASA classification, lesion location, stenosis degree, body mass index and procedure duration

*COT* conventional oxygen therapy; *NFJV* normal frequency jet ventilation; *HFJV* high frequency jet ventilation

Lesions location: 1) the upper and middle part of the main airway, 2) the middle and lower part of the main airway, 3) the left or right main bronchial and 4) in the distal bronchial. Degree of stenosis: 1 for 0–59%, 2 for 60–89%, and 3 for above 90%

**Table 6:** Multiple linear regression analysis of blood gas PaCO_2_ related factors

Variables	Regression coefficient	95% Confidence interval	*P* Value
Body mass index	0.3	(−0.4, 1.0)	0.425
Procedure duration	0.1	(−0.1, 0.3)	0.438
Group COT/NFJV/HFJV	0.2	(−2.6, 2.9)	0.905
Lesion location	−0.4	(−2.5, 1.7)	0.714
Degree of stenosis	−2.0	(−4.9, 1.0)	0.194

The above indexes are not independent influencing factors of blood gas PaCO_2_

*COT* conventional oxygen therapy; *NFJV* normal frequency jet ventilation; *HFJV* high frequency jet ventilation

Lesions location: 1) the upper and middle part of the main airway, 2) the middle and lower part of the main airway, 3) the left or right main bronchial and 4) in the distal bronchial. Degree of stenosis: 1 for 0–59%, 2 for 60–89%, and 3 for above 90%

## Discussion

BI has shown remarkable advancements in pulmonary medicine diagnostics and therapy in recent years. Monitored anesthesia care (MAC) is performed in basic and some advanced BI [11]. By maintaining spontaneous breathing, without the need for LMA or intubation, MAC could meet the surgical needs and alleviates the patients’ discomfort [1]. The commonly used ventilation modes included COT, intermittent ventilation, controlled mechanical ventilation, and jet ventilation (manual or automatic, high or normal frequency) [11, 12]. Ventilation mode is the most direct factor affecting the oxygenation of patients, especially in MAC [6, 13]. In the present study, endoscopic face mask and three ventilation modes were used to provide oxygen to patients [14].

HFJV has become a technique for maintaining ventilation developed by Klain and Smith [5, 6]. Gas enters the breathing path at low pressure through a narrow jet tube. Open system, high frequency and low tide are the three characteristics of HFJV. This technique has become one of the most important ventilation modes in airway management, especially in the ASA guidelines for interventional pulmonology procedures [7]. In the present study, the effect of oxygenation maintenance in COT, NFJV, and HFJV was compared via face mask during BI under MAC. The PaO_2_ in the HFJV group increased to 251.7 mmHg, higher than that in the COT and NFJV groups. HFJV has certain PEEP effects, which can open the airway, reduce the anatomical inefficiency of the nasopharyngeal cavity, improve the final expiratory volume of the lung, and increase the effective ventilation of the alveoli [15, 16]. The PaO_2_ in the NFJV group was 192.0 mmHg, higher than that in the COT group (176.3 mmHg), but no statistical difference was found. The mechanism of NFJV was similar with that of HFJV. In addition, NFJV may cause asynchronous breathing, resulting in less significant oxygenation improvement than that in HFJV. The results indicated that HFJV is an effective and safe oxygen supplementation for patients under MAC in BI.

In addition to PaO_2_, another point of concern was the patient’s PaCO_2_. A previous review proved that NFJV was more suitable for BI than HFJV, which may cause CO_2_ accumulation when jet frequency was above 150/min [17]. The PaCO_2_ values in the COT, NFJV, and HFJV groups were 59.2 mmHg, 59.8 mmHg and 59.6 mmHg, respectively, and no significant difference was found among them. Although the PaCO_2_ value was higher than the upper limit of the normal value, no delayed awakening occurred. Pearson analysis showed no important factors affecting PaCO_2_ and certainly no independent factors, either. Mild hypercapnia may not cause brain injury and only worsen the condition of the brain when PaCO_2_ is above 100 mmHg [8, 18, 19]. Thus anesthesiologists did not provide any treatment when the PaCO_2_ was a little above 50 mmHg.

Intraoperative oxygenation of patients might be affected by many factors. In this study, multiple linear regression showed that only BMI and procedure duration were independent influencing factors of PaO_2_, and there was no independent influencing factors of PaCO_2_. The key of MAC in BI is how to maintain the patients’ oxygenation in open airway and ensure the feasibility of the procedures. Pathological airways often cause restricted ventilation dysfunction, resulting in decreased respiratory power, increased elastic resistance, decreased alveolar compliance, and limited lung expansion, which are all important factors affecting PaO_2_ [4]. Meanwhile, the extent and location of the lesions as well as whether the open airway aggravates its effect on PaO_2_, are problems worth solving. In the present study, the location of airway lesions (which was divided into four types) and the severity of airway stenosis (which was graded into three levels), were speculated to be the important factors related to PaO_2_. However, Pearson analysis showed that both were not important factors affecting PaO_2_, and certainly were not independent factors.

In this research, the incidence of adverse events was lower than that previous reported [10, 20]. Among the 150 patients, only seven developed intra-operative hypoxemia, which was relatively short and the oxygenation was effectively improved after treatment. Hypoxemia mainly occurred at two stages: one was after induction of anesthesia and the other was in the period of balloon dilation. Rapid anesthesia induction may lead to respiratory depression, as manifested by decreasing RR and tidal volume, the absence of spontaneous breathing and even the occurrence of apnea. SpO_2_ was effectively returned to normal after the anesthesiologist reduced the depth of anesthesia to restore spontaneous breathing or with the assistance of bag-mask ventilation. Balloon dilatation resulted in transient complete airway obstruction. After balloon dilatation, normal ventilation could be restored, and SpO_2_ could quickly return to normal [21, 22].

Many deficiencies in this study deserve further research. First, the dose and speed of anesthetic administration during MAC determined the depth of anesthesia and the degree of respiratory depression which determined PaO_2_ directly. No statistically significant difference was found in the anesthetic doses among the three groups. However, PaO_2_ may still be affected due to the differences in the patients’ conditions and the operating habits of the anesthesiologists. Second, the ABG collection time was set at 15 min after the operation, when the patient was basically in a stable state of anesthesia. At this time, the airway obstruction was mostly treated already. Finally, only the sedation score of 3–4 was recorded. Bispectral index and neuromonitoring were not performed.

## Conclusion

Compared with COT and NFJV, HFJV via endoscopic face mask could improve the intra-operative PaO_2_ in patients with airway stenosis more effectively and safely during basic and some advanced BI in deep sedation. PaO_2_ was correlated with ventilation mode, BMI and procedure duration. BMI and procedure duration were independent influencing factors of arterial blood gas PaO_2_. PaCO_2_ was not correlated with ventilation mode and other preoperative factors, which were not independent influencing factors either.

## Data Availability

The datasets used and/or analyzed during the current study are available from the corresponding author.

## Abbreviations

COT: Conventional oxygen therapy
NFJV: Normal frequency jet ventilation
HFJV: High frequency jet ventilation
BI: Bronchoscopic intervention
RR: Respiratory rate
LMA: Laryngeal mask airway
SJV: Supraglottic jet ventilation
ECG: Electrocardiogram
SpO_2_: Pulse oximetry
RSS: Ramsey Sedation Scale
FiO_2_: Fractional inspired oxygen
DP: Driving pressure
MBP: Mean blood pressure
HR: Heart rate
ABG: Arterial blood gas
ANOVA: Univariate analysis of variance
LSD: Least significance difference
BMI: Body mass index
MAC: Monitored anesthesia care

## References

[1] Galway U, Zura A, Khanna S, Wang M, Turan A, Ruetzler K (2019). Anesthetic considerations for bronchoscopic procedures: a narrative review based on the Cleveland Clinic experience. J Thorac Dis.

[2] Krecmerova M, Schutzner J, Michalek P, Johnson P, Vymazal T (2018). Laryngeal mask for airway management in open tracheal surgery-a retrospective analysis of 54 cases. J Thorac Dis.

[3] Thiruvenkatarajan V, Dharmalingam A, Arenas G, Wahba M, Steiner R, Kadam VR, Tran A, Currie J, Van Wijk R, Quail A (2020). High-flow nasal cannula versus standard oxygen therapy assisting sedation during endoscopic retrograde cholangiopancreatography in high risk cases (OTHER): study protocol of a randomised multicentric trial. Trials.

[4] Zhao H, Wang H, Sun F, Lyu S, An Y (2017). High-flow nasal cannula oxygen therapy is superior to conventional oxygen therapy but not to noninvasive mechanical ventilation on intubation rate: a systematic review and meta-analysis. Crit Care.

[5] Galmen K, Harbut P, Freedman J, Jakobsson JG (2017). The use of high-frequency ventilation during general anaesthesia: an update. F1000Research.

[6] Hohenforst-Schmidt W, Zarogoulidis P, Huang H, Man YG, Laskou S, Koulouris C, Giannakidis D, Mantalobas S, Florou MC, Amaniti A (2018). A new and safe mode of ventilation for interventional pulmonary medicine: the ease of nasal superimposed high frequency jet ventilation. J Cancer.

[7] de Lima A, Kheir F, Majid A, Pawlowski J (2018). Anesthesia for interventional pulmonology procedures: a review of advanced diagnostic and therapeutic bronchoscopy. Can J Anaesth.

[8] Cheng Q, Zhang J, Wang H, Zhang R, Yue Y, Li L (2015). Effect of acute Hypercapnia on outcomes and predictive risk factors for complications among patients receiving Bronchoscopic interventions under general anesthesia. PLoS One.

[9] Whittle JS, Pavlov I, Sacchetti AD, Atwood C, Rosenberg MS (2020). Respiratory support for adult patients with COVID-19. J Am Col Emerge Phys Open.

[10] Liang H, Hou Y, Sun L, Li Q, Wei H, Feng Y (2019). Supraglottic jet oxygenation and ventilation for obese patients under intravenous anesthesia during hysteroscopy: a randomized controlled clinical trial. BMC Anesthesiol.

[11] Chadha M, Kulshrestha M, Biyani A (2015). Anaesthesia for bronchoscopy. Indian J Anaesthesia.

[12] Fadaizadeh L, Hoseini MS, Bagheri M (2014). Anaesthesia management during interventional Bronchoscopic procedures: laryngeal mask airway or rigid bronchoscope. Turkish J Anaesthesiol Reanimation.

[13] Fuehner T, Fuge J, Jungen M, Buck A, Suhling H, Welte T, Gottlieb J, Greer M (2016). Topical nasal anesthesia in flexible bronchoscopy--a cross-over comparison between two devices. PLoS One.

[14] Nisi F, Galzerano A, Cicchitto G, Puma F, Peduto VA (2015). Improving patient safety after rigid bronchoscopy in adults: laryngeal mask airway versus face mask - a pilot study. Med Devices.

[15] Helviz Y, Einav S (2018). A systematic review of the high-flow nasal cannula for adult patients. Crit Care.

[16] Bialka S, Copik M, Rybczyk K, Owczarek A, Jedrusik E, Czyzewski D, Filipowski M, Rivas E, Ruetzler K, Szarpak L (2018). Assessment of changes of regional ventilation distribution in the lung tissue depending on the driving pressure applied during high frequency jet ventilation. BMC Anesthesiol.

[17] Putz L, Mayne A, Dincq AS (2016). Jet ventilation during rigid bronchoscopy in adults: a focused review. Biomed Res Int.

[18] Cheng Q, Li L, Yang M, Sun L, Li R, Huang R, Ma J (2019). Moderate hypercapnia may not contribute to postoperative delirium in patients undergoing bronchoscopic intervention. Medicine.

[19] Cheng Q, Li L, Lin D, Li R, Yue Y, Wei H, Ma J (2019). Effects of acute hypercapnia on cognitive function in patients undergoing bronchoscope intervention. J Thorac Dis.

[20] Chung SM, Choi JW, Lee YS, Choi JH, Oh JY, Min KH, Hur GY, Lee SY, Shim JJ, Kang KH (2019). Clinical effectiveness of high-flow nasal cannula in Hypoxaemic patients during Bronchoscopic procedures. Tuberculosis Respiratory Dis.

[21] Pawlowski J (2013). Anesthetic considerations for interventional pulmonary procedures. Curr Opin Anaesthesiol.

[22] Sarkiss M (2011). Anesthesia for bronchoscopy and interventional pulmonology: from moderate sedation to jet ventilation. Curr Opin Pulm Med.

